# Supplementary material for: The causal relationship between the human gut microbiota and pyogenic arthritis: a Mendelian randomization study
Source: Front Cell Infect Microbiol. 2024 Nov 26;14:1452480. doi: 10.3389/fcimb.2024.1452480 (PMC11629706; doi:10.3389/fcimb.2024.1452480)
Supplement: Supplementary file 1 [file DataSheet1.zip › mendelian test/ebi-a-GCST90027518.csv_forest.pdf]

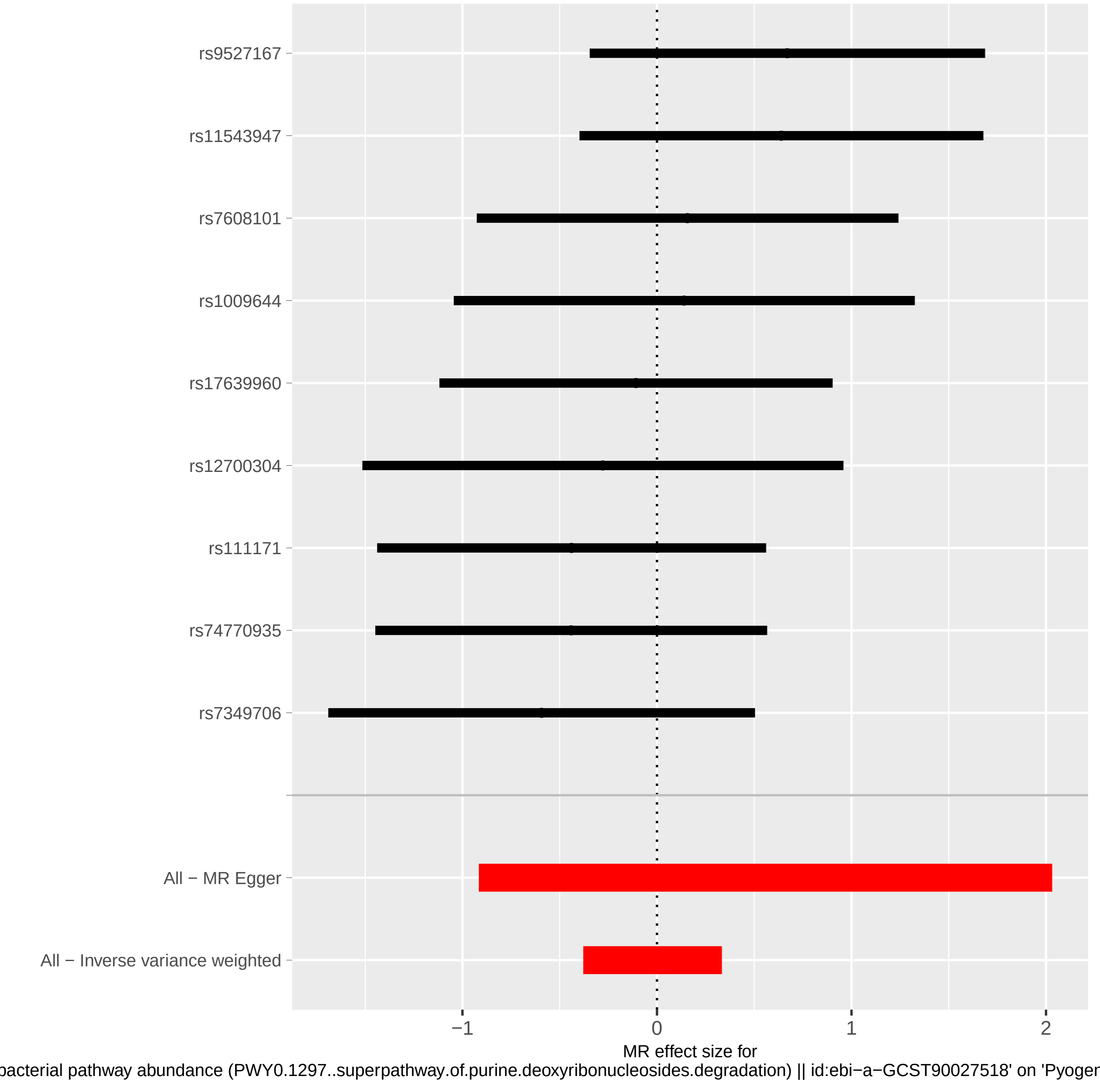
rs9527167
rs11543947
rs7608101
rs1009644
rs17639960
rs12700304
rs111171
rs74770935
rs7349706
All − MR Egger
All − Inverse variance weighted
−1
0
1
2
MR effect size for
bacterial pathway abundance (PWY0.1297..superpathway.of.purine.deoxyribonucleosides.degradation) || id:ebi−a−GCST90027518' on 'Pyogen
